# Effects of smoking cessation on serum leptin and adiponectin levels

**DOI:** 10.1186/s12971-015-0054-7

**Published:** 2015-09-03

**Authors:** Maria Kryfti, Katerina Dimakou, Michail Toumbis, Zoe Daniil, Chryssi Hatzoglou, Konstantinos I. Gourgoulianis

**Affiliations:** 6th Respiratory Medicine Department, Sotiria General Hospital for Thoracic Diseases, Mesogeion 152, 11527 Athens, Greece; Respiratory Medicine Department, University of Thessaly School of Medicine, University Hospital of Larissa, Larissa, 41110 Greece

**Keywords:** Leptin, Adiponectin, Smoking, Cessation, Tobacco

## Abstract

**Background:**

Evidence on the association of leptin and adiponectin and smoking is limited and discordant. Leptin and adiponectin represent the most abundant adipokines in human plasma that play crucial roles in the pathophysiology of metabolic syndrome, atherosclerosis and insulin resistance. Leptin up-regulates the expression of several pro-inflammatory cytokines and is increased upon weight gain. Adiponectin has been shown to possess insulin sensitizing, anti -inflammatory and anti-atherogenic properties and is increased upon weight reduction. Our aim was to assess the effects of smoking cessation on serum leptin and adiponectin levels.

**Methods:**

We assessed the changes in serum leptin and adiponectin levels, serum CRP levels and BMI in apparently healthy smokers after 3 and 6 months of abstinence from smoking. Successful cessation was confirmed by an exhaled carbon monoxide measurement. 26 healthy non-smokers were recruited as controls.

**Results:**

Among the sample group, 32 subjects had quitted smoking at 3 months and 29 subjects at 6 months. Samples’ leptin increased significantly from baseline to three months (mean change 3.76 ng/ml [95 % CI 0.89, 6.64], p =0.012) and then decreased significantly from three to six months of smoking cessation (mean change -4,29 ng/ml [95 % CI −7.34, −6.64], *p* = 0.008). Samples’ adiponectin increased significantly from baseline to three months of abstinence from smoking (mean change 2.34 [95 % CI −0.05, 4.73], p −0.05). BMI was significantly increased (mean change 2.03 kg/m^2^ [95 % CI 1.60, 2.46], p <0.05), while CRP decreased significantly from baseline to 6 months of smoking cessation (mean change −0.68 mg/dl [95 % CI −1.06, −0.30], *p* = 0.001).

**Conclusions:**

Smoking quitters’ leptin levels appear to increase 3 months after smoking cessation and then decrease from 3 to 6 months of abstinence from smoking. Adiponectin levels increase during the first trimester of smoking cessation. The decrease in CRP levels indicates that the low grade inflammation observed in smokers is gradually restored. The alterations of serum leptin and adiponectin after 6 months of smoking cessation suggest the same but do not reach statistically significant levels. Weight gain and changes in fat distribution may attenuate the beneficial effects of smoking cessation.

## Background

Smoking is a major atherosclerotic risk factor and is associated with the development of metabolic syndrome leading to cardiovascular disease [1–3]. Among other harmful effects, long term smoking is reported to increase inflammation, lipid peroxidation, endothelial cell dysfunction and insulin resistance [4–6]. Since it is evident that adiponectin protects and leptin accelerates the development of atherosclerotic diseases we can speculate that these adipokines may be the link between smoking and cardiovascular and metabolic diseases.

Leptin and adiponectin are secreted by adipose tissue and represent the most abundant adipokines in human plasma. Since its cloning in 1994 [7], leptin has been acknowledged as a major endocrine signal in the homeostatic control of body weight [8]. Weight gain is associated with increased circulating leptin levels while fasting reduces leptin levels [9]. Beyond its metabolic functions, leptin is a pleiotropic cytokine involved in the recruitment, activation and survival of inflammatory cells [10]. On the other hand, adiponectin, an adipocyte-derived protein, has been shown to possess insulin sensitizing, anti-inflammatory and anti-atherogenic properties [8, 11, 12]. Furthermore, adiponectin concentration can be upregulated upon weight reduction [13].

Both cigarette smoking and obesity are accompanied by a low grade subclinical chronic inflammation. Leptin and adiponectin can be considered markers of subclinical inflammation with opposing effects and it is well appreciated that plasma levels of C-reactive protein have a positive correlation with plasma leptin and a negative correlation with plasma adiponectin [14–16]. Despite the well known inverse association between smoking and body weight, there have been conflicting reports on the effects of smoking on serum leptin and adiponectin levels [17–22].

The aim of this study is to examine the effects of smoking cessation on circulating leptin and adiponectin levels.

## Methods

### Selection criteria

The patients included in our study were recruited from the outpatient smoking cessation department of “Sotiria General Chest Hospital”. Eligibility criteria included age ≥18 years, smoking of ≥15 cigarettes/day for ≥5 years, and self-motivation for quitting. We excluded patients with a history of diabetes mellitus, severe obesity or cachexia, chronic alcohol abuse, acute infection such as respiratory tract infection or COPD exacerbation one month prior to the study, chronic inflammatory diseases such as asthma or interstitial lung disease, collagen vascular disease or disturbances of thyroid function and chronic diseases such as heart failure, chronic renal failure and lung cancer. All the above mentioned conditions are reported by several studies to affect serum leptin, adiponectin and CRP levels and could have a confounding effect regarding our results [23–28]. Healthy registrars and consultants employed at “Sotiria General Chest Hospital” were recruited as controls. The subjects included in the control group were lifelong non-smokers. The exclusion criteria were the same as the ones applied for the sample group. The enrolment of the sample group took place from September 2011 until December 2012. The controls were recruited at the same time as the sample group. All the participants signed a consent form. Our study was approved by the Ethics Committee of University of Larissa and our institution.

### Intervention and measurements

Baseline characteristics of all patients were recorded prior to commencing the smoking cessation program (Table 1). Nicotine dependence was measured via the eight-item Fagerstrom Tolerance Questionnaire [29]. Cumulative smoking exposure was determined in terms of pack-years by multiplying the number of years smoked with the average number of packs per day . The patients had the option of a pharmacological approach or a non-pharmacological approach to quit smoking. The patients who decided to take pharmaceutical aid were administered bupropion or varenicline as anti-smoking drugs according to their medical history and nicotine dependence.

**Table 1:** Samples’ characteristics at baseline and 3 months and 6 months after smoking cessation

Participants’ characteristics	Baseline	3 months	6 months
Age (years)^a^	56.1 ± 9.4	–	–
Female sex^a^	16 (45.7)	–	–
BMI (kg/m^2^)	27.1 ± 3.7	28.6 ± 3.6^*^	29.2 ± 3.5^*,**^
Smoking (pack-years)^a^	54.3 ± 26.7	–	–
Exhaled CO	14.7 ± 9.4	4.3 ± 2.5^*^	4.0 ± 2.7^*^
Serum leptin (ng/ml)	6.9 ± 3.1	10.5 ± 6.8^*^	6.5 ± 5.2^**^
Serum adiponectin (μg/ml)	9.5 ± 2.8	11.8 ± 5.5^*^	9.6 ± 5.2
CRP (mg/dl)	0.8 ± 1.1	0.3 ± 0.3^*^	0.3 ± 0.3^*^

Data are expressed as number of subjects (%) or mean value ± standard deviation. Differences not marked with one of the above symbols are not significant

^*^ *p* < 0.05 vs. baseline

^**^ *p* < 0.05 vs. 3 months

^a^Assessment applicable only at baseline

The subjects who successfully quit smoking within the time limits of our study constituted the sample group and healthy non-smokers constituted the control group. Successful cessation was confirmed by an exhaled carbon monoxide level below 8 ppb, a cut-off providing 90 % sensitivity and specificity for detecting tobacco use [30]. Blood samples were obtained in the morning following an overnight fasting for determination of serum adiponectin, leptin and CRP before study entry and at 3,6 months after smoking cessation. The blood samples were collected at the same time in the morning both for samples and controls at each visit and then stored at −80 °C until biochemical assay. Serum concentration of leptin and adiponectin were measured by a commercially available sandwich enzyme-linked immunosorbent assay (ELISA, Raybiotec Inc.) with detection limit 7.8 pg/mL for leptin and 0.246 ng/mL for adiponectin. Serum CRP was measured by high-sensitivity CRP assay. Body mass index was also calculated before study entry and at the end of month 3 and 6.

### Statistical analysis

We performed power analysis using as reference values the levels of leptin and adiponectin reported by several studies in smokers, ex-smokers and non-smokers [31, 32]. A sample size of ≥32 subjects was calculated to be adequate for detecting half of a standard deviation (1.5 mg/L) of basal leptin and adiponectin with a power 80 % at a significance level of 5 % via two-tailed tests. A starting number of participants of ≥160 was calculated on the assumption that 20 % of the participants manage to quit.

The Pearson correlation coefficient was used to describe the relationships between variables. The Student’s *t* test for continuous paired & unpaired data was used for intra and inters group comparisons. The correlation-coefficient *p*-values where calculated based on the Pearson’s product moment correlation coefficient. Multiple regression analysis was performed using a Generalized Linear Model. Model selection for the multiple regression analysis was performed by utilizing a bi-directional stepwise algorithm.

## Results

One hundred sixty eight apparently healthy smokers of both sexes were recruited. Adequate follow up data were available for 162 individuals. Among the 162 enrolled subjects, 36 individuals successfully quit smoking for 3 months and 29 of them reached 6 months of smoking cessation. Four of the subjects who successfully quit smoking for 6 months did not visit the clinic for their first evaluation at 3 months, so data were available only for 32 individuals at 3 months. Only data from the subjects who successfully quit smoking were analyzed. Ten individuals used varenicline and 22 stopped smoking without pharmaceutical aid. Three individuals completed the study after discontinuing varenicline . The median interval between study entry and actual quitting time was 6 days (range 2–22 days). The measurement of exhaled carbon monoxide levels was performed after a median interval of 92 days (range 85–98 days). The control group included 26 healthy non-smokers.

Samples’ leptin increased significantly from baseline to three months of abstinence from smoking (mean change 3.76 ng/ml [95 % CI 0.89, 6.64], *p* = 0.012, Table 1). A statistically significant decline in samples’ leptin was observed from three to six months of smoking cessation (mean change −4.29 ng/ml [95 % CI −7.34, −6.64], *p* = 0.008, Table 1). Although serum leptin levels decreased from baseline to 6 months of abstinence from smoking, this decline was not statistically significant (*p* > 0.05). Controls’ leptin did not change from baseline to six months (*p* > 0.05, Table 2).

**Table 2:** Controls’ characteristics at baseline and after 6 months

Participants’ characteristics	Baseline	6 months
Age (years)^a^	33.7 ± 6.0	–
Female sex^a^	22 (84.6)	–
BMI (kg/m^2^)	23.1 ± 5.1	23.2 ± 5.1
Smoking (pack-years)^a^	54.3 ± 26.7	–
Exhaled CO	3.5 ± 2.2	3.7 ± 1.9
Serum leptin (ng/ml)	2.1 ± 1.4	2.1 ± 1.4
Serum adiponectin (μg/ml)	1.5 ± 1.3	1.5 ± 1.2
CRP (mg/dl)	0.06 ± 0.06	0.05 ± 0.05

Data are expressed as number of subjects (%) or mean value ± standard deviation. Differences not marked with one of the above symbols are not significant

^a^Assessment applicable only at baseline

Samples’ adiponectin increased significantly from baseline to three months after smoking cessation (mean change 2.34 μg/ml [95 % CI −0.05, 4.73], *p* = 0.05, Table 1). From three to six months of abstinence from smoking adiponectin levels decreased (*p* > 0.05). Serum adiponectin levels increased from baseline to six months of smoking cessation but this rise did not reach statistically significant levels. Controls’ adiponectin changes from baseline to six months were not significant (*p* > 0.05, Table 2).

Samples’ BMI increased significantly from baseline to 6 months after quitting smoking (mean change 2.03 kg/m^2^ [95 % CI 1.60, 2.46], *p* = 2.7E-010, Table 1), whereas controls’ BMI did not change from baseline (*p* > 0.05, Table 2). Following smoking cessation, BMI increased at 3 months (mean change 1.45 kg/m^2^ [95 % CI 1.18, 1.72], *p* = 5.71E-012) and this increase remained significant from 3 to 6 months in the sample group (mean change 0.55 kg/m^2^ [95 % CI 0.12, 0.97], *p* = 0.014).

CRP decreased significantly from baseline to 3 months (mean change −0.54 mg/dl [95 % CI −0.95, −0.14], *p* = 0.01, Table 1) and from baseline to 6 months of abstinence from smoking (mean change −0.68 mg/dl [95 % CI −1.06, −0.30], *p* = 0.001, Table 1). The controls’ CRP levels remained stable (*p* > 0.05, Table 2).

**Fig. 1:**
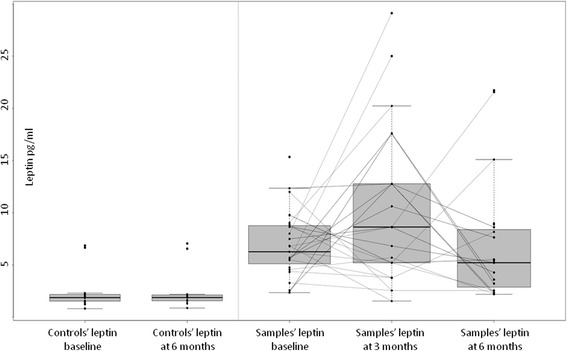
Scatterplot of samples and controls’ leptin at baseline and after smoking cessation

**Fig. 2:**
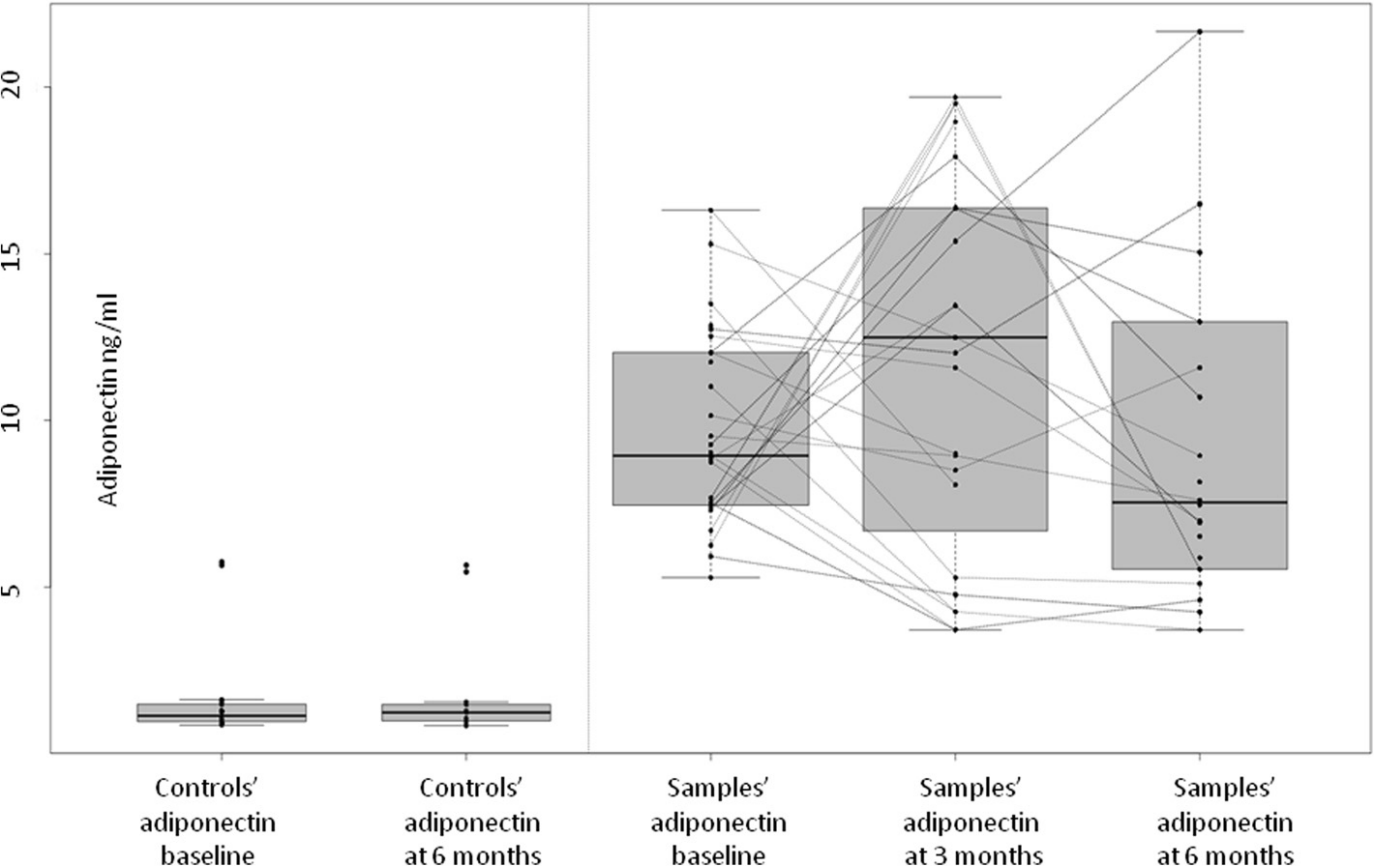
Scatterplot of samples’ and controls’ adiponectin at baseline and after smoking cessation

Intra-individual changes of leptin and adiponectin are illustrated in Fig. 1 and Fig. 2.

Tables 3 and 4 demonstrate linear relationships between adiponectin and leptin and participants’ characteristics in the overall population at month 6. There was a strong positive association between age and adiponectin, while leptin exhibited a negative correlation with age.

**Table 3:** Linear relationships between adiponectin and participants’ characteristics in the total population at month 6

Variable	Correlation coefficient r	*p* value
Age^a^	0.589	<0.001
Gender (male = 1, female = 2)^a^	0.320	0.097
Smoking (pack/years)^a^	0.142	0.470
Exhaled Carbon Monoxide^a^	−0.328	0.089
Body mass index	−0.026	0.899
CRP	0.260	0.181
Leptin	−0.153	0.437

^a^Assessment at baseline

**Table 4:** Linear relationships between leptin and participants’ characteristics in the total population at month 6

Variable	Correlation coefficient r	*p* value
Age^a^	−0.374	0.050
Gender (male = 1, female = 2)^a^	−0.303	0.117
Smoking (pack/years)^a^	−0.173	0.378
Exhaled Carbon Monoxide^a^	0.146	0.457
Body mass index	0.255	0.165
CRP	0.033	0.867
Leptin	−0.153	0.437

^a^Assessment at baseline

We performed multiple regression analysis to examine whether leptin and adiponectin levels variation could be explained by specific independent predictors of leptin and adiponectin including age, gender, BMI and smoking (Tables 5 and 6). A multiple regression model including the difference of leptin levels between baseline and third month, the gender and the log-transformed age parameter explained ~66 % of the adiponectin levels variation at 6 months (adjusted R^2^ = 0.66). The analysis also produced a statistically significant model for leptin at 6 months containing only the difference of adiponectin levels between baseline and the third month which explained 16 % of leptin levels variation (adjusted R^2^ = 0.16).

**Table 5:** Independent predictors of adiponectin levels in the total population at month 6

Variable	Standardized β coefficient	Standard error	*p* value
Leptin 0 - Leptin 3^a^	−0.025	0.012	0.042
Gender	0.526	0.138	0.001
Log (Age)	3.095	0.480	<0.001

^a^Difference of leptin levels at 3 months from leptin levels at baseline

**Table 6:** Independent predictors of leptin levels in the total population at month 6

Variable	Standardized β coefficient	Standard error	*p* value
Adiponectin 0 - Adiponectin 3^a^	−0.025	0.012	0.042

^a^Difference of adiponectin levels at 3 months from adiponectin levels at baseline

## Discussion

Our study showed smoking quitters’ leptin levels to increase significantly 3 months after smoking cessation and then decrease significantly from 3 to 6 months of abstinence from smoking. Adiponectin levels increased significantly during the first trimester of smoking cessation. These alterations of serum leptin and adiponectin concentration occurred along with a significant increase in BMI and a significant decrease in CRP levels after 6 months of abstinence from smoking.

Only a few studies addressed the impact of quitting smoking on leptin concentration. Eliasson and Smith suggested that the leptin levels would increase 8 weeks after smoking cessation [17]. Perkin and Fonte also reported that the leptin levels increased, particularly in female smokers 3 weeks after nicotine abstinence [18]. However, Nicklas et al. suggested that the changes observed in leptin concentration in 13 patients after 6 months of smoking cessation were not significant [19]. A significant increase in serum leptin levels one year after smoking cessation was reported in a recent study by Gonseth et al. [22].

The elevated plasma leptin concentration observed in smokers may be due to an increase in adipose tissue secretion of leptin or a decrease in leptin clearance. Previous studies report that nicotine increases adipose tissue lipolysis [33, 34], and smokers have elevated fasting adipose tissue lipoprotein lipase activity [35, 36]. Cigarette smoking enhances the adrenal release of glucocorticoids [37], which elevate plasma leptin concentrations by increasing leptin expression in adipose tissue [38].

In our study the increased leptin levels 3 months after smoking cessation occurred after a preceding increase in body weight and may reflect the increase in body fat after smoking cessation. The fact that leptin initially increases may imply that leptin was influenced by the increase in body weight predominantly the first trimester of smoking cessation. From 3 to 6 months of abstinence from smoking, leptin levels decrease despite weight gain. If we consider the role of leptin in the recruitment, activation and survival of inflammatory cells we can assume that this decline in leptin levels indicates that the low grade subclinical inflammation observed in smokers is gradually restored after smoking cessation.

In contrast to the findings that weight reduction is associated with an increase in adiponectin concentration [13, 39] we demonstrated that serum adiponectin concentration increased three months after smoking cessation despite of weight gain. This is in line with several previous studies [20, 21]. Otsuka et al. reported that plasma adiponectin levels in Japanese patients were elevated 6 months after smoking cessation [20]. Efstathiou et al. also demonstrated that adiponectin levels in Greek smokers increased 9 weeks after nicotine abstinence [21].

Several explanations have been proposed for the mechanisms by which smoking cessation modulates adiponectin expression and secretion. Smoking provokes oxidative stress and inflammatory cytokines that reduce the expression and secretion of adiponectin [40]. Nicotine itself induces lipolysis and may suppress adiponectin gene expression [40, 41]. Adiponectin accumulates in the injured vascular walls of smokers. Therefore, the increased consumption of circulating adiponectin might represent another mechanism causing lower levels of adiponectin in smokers [42].

Both smoking and body weight affect adiponectin levels. During the first trimester of smoking cessation adiponectin levels increased significantly in the sample group despite weight gain. Although adiponectin levels finally increase from baseline to 6 months of abstinence from smoking, this increase does not reach statistically significant levels. It is possible that during the second trimester of smoking cessation the adiponectin decreasing effect of weight gain towers above the adiponectin increasing effect of smoking cessation.

BMI increased significantly during the period of abstinence in this study. It is well proven that cigarette smoking is associated with lower body weight. Cross-sectional studies show that smokers weigh less than age-matched non-smokers, while longitudinal data show that most smokers gain weight after smoking cessation [35, 43, 44]. Several studies reported that smoking cessation reversed the increased energy expenditure in smokers, and the BMI of the quitters increased [18, 44, 45]. However, the specific mechanisms by which smoking affects body weight are not completely clear. It has been reported that weight gain is a result of increased food consumption and decreased energy expenditure upon cessation of smoking [46–49]. Smoking increases the adrenergic activity, which can directly increase thermogenesis and reduce weight. In addition, it seems that nicotine has a direct effect on adipose tissue metabolism during smoking which influences the rate of weight gain after smoking cessation [33, 35, 36].

We did not detect a positive or a negative correlation of leptin or adiponectin with BMI. A possible explanation is that changes in leptin and adiponectin are associated with changes in fat distribution after smoking cessation. The degree of obesity as measured by BMI may not reflect the amount and distribution of adipose tissue.

Similar to previous reports, CRP plasma levels decreased significantly from baseline to 6 months after smoking cessation [50]. Most studies that have examined CRP status in former smokers suggest that levels fail to fall immediately upon cessation, which reflects the fact that the underlying tissue damage caused by smoking takes some time to recover [50]. CRP is a well known marker of subclinical inflammation and therefore the decrease in CRP levels observed in our study indicates that low grade inflammation is gradually restored after smoking cessation.

Multivariate analysis revealed an interesting association between leptin and adiponectin. It appears that the difference of either one of these two adipokines from baseline to 3 months of smoking abstinence is predictive for the levels of the other adipokine 6 months after smoking cessation. It is likely that leptin and adiponectin act via several interrelated metabolic pathways. Smoking seems to change the balance between these two adipokines causing effects on inflammatory processes and insulin resistance that may contribute to the pathogenesis of cardiovascular disease [12, 51–53].

Several methodological aspects of our study require consideration. Although quitters’ exhaled CO levels were compatible with moderate exposure to environmental tobacco smoke this could not elucidate the effects of passive smoking on serum leptin and adiponectin levels. Furthermore, the possibility of any direct impact of varenicline on serum leptin and adiponectin levels could not be excluded. Although the BMI is a reasonable measure of adiposity, it may not always precisely reflect the quantity of body fat [20]. The present findings could be altered if the amount and distribution of adipose tissue were obtained by measuring waist and hip circumference or other measures of total and abdominal adiposity in place of BMI. Another limitation of the study is not measuring triglycerides and HDL-cholesterol that could independently modulate leptin and adiponectin levels [54]. Having in mind that hypadiponectinemia in overweight individuals and in type II diabetic patients is closely associated with insulin resistance and hyperinsulinemia [55], we excluded subjects with type II diabetes and severe obesity. Nevertheless, the lack of insulin data must be considered a limitation of our study since it has been suggested recently that insulin may be involved in the association of smoking and leptin [22]. Further research on this subject is needed.

Previous reports showed that smoking-related damage via low grade inflammation and endothelial dysfunction may persist after smoking cessation and continue to influence leptin and adiponectin levels [56]. Thus, larger and durable cohort studies are required to assess whether the alterations observed in serum leptin and adiponectin concentration after abstinence from smoking exert a protective effect against cardiovascular disease and metabolic diseases.

## Conclusions

Smoking quitters’ leptin levels appear to increase 3 months after smoking cessation and then decrease from 3 to 6 months of abstinence from smoking. Adiponectin levels increase during the first trimester of smoking cessation. The decrease in CRP levels indicates that the low grade inflammation observed in smokers is gradually restored. The alterations of serum leptin and adiponectin after 6 months of smoking cessation suggest the same but do not reach statistically significant levels. Weight gain and changes in fat distribution may attenuate the beneficial effects of smoking cessation.
